# Landmark-Assisted Anatomy-Sensitive Retinal Vessel Segmentation Network

**DOI:** 10.3390/diagnostics13132260

**Published:** 2023-07-04

**Authors:** Haifeng Zhang, Yunlong Qiu, Chonghui Song, Jiale Li

**Affiliations:** College of Information Science and Engineering, Northeastern University, Shenyang 110819, China; haif_zh@163.com (H.Z.); qyl6016@163.com (Y.Q.); li_jiale2020@163.com (J.L.)

**Keywords:** retinal vessel segmentation, TransUNet self-supervised landmark, contrastive learning

## Abstract

Automatic retinal vessel segmentation is important for assisting clinicians in diagnosing ophthalmic diseases. The existing deep learning methods remain constrained in instance connectivity and thin vessel detection. To this end, we propose a novel anatomy-sensitive retinal vessel segmentation framework to preserve instance connectivity and improve the segmentation accuracy of thin vessels. This framework uses TransUNet as its backbone and utilizes self-supervised extracted landmarks to guide network learning. TransUNet is designed to simultaneously benefit from the advantages of convolutional and multi-head attention mechanisms in extracting local features and modeling global dependencies. In particular, we introduce contrastive learning-based self-supervised extraction anatomical landmarks to guide the model to focus on learning the morphological information of retinal vessels. We evaluated the proposed method on three public datasets: DRIVE, CHASE-DB1, and STARE. Our method demonstrates promising results on the DRIVE and CHASE-DB1 datasets, outperforming state-of-the-art methods by improving the F1 scores by 0.36% and 0.31%, respectively. On the STARE dataset, our method achieves results close to the best-performing methods. Visualizations of the results highlight the potential of our method in maintaining topological continuity and identifying thin blood vessels. Furthermore, we conducted a series of ablation experiments to validate the effectiveness of each module in our model and considered the impact of image resolution on the results.

## 1. Introduction

Retinal vessel segmentation is an important diagnostic method for detecting hypertension, arteriosclerosis, and retinal diseases [1]. However, the retinal vascular structure is extremely complex, and the distribution of vascular pixel intensity is unbalanced. Furthermore, due to the low contrast between the blood vessel pixels and the background, the thin blood vessels located at the ends of the vascular structures are difficult to completely segment from the background. Accurate retinal vessel segmentation has always been an extremely challenging task.

In recent years, a great deal of work has focused on automatically segmenting retinal blood vessels. The methods used are broadly classified into two groups: unsupervised and supervised methods. Unsupervised methods are suitable for image segmentation with little annotation information. Commonly used algorithms include the matched filtering method [2], multi-threshold blood vessel detection method [3], mathematical morphology method [4], and so on. However, due to the absence of supervision from prior knowledge, unsupervised methods can easily detect false edges and achieve lower performance. In contrast to unsupervised methods, supervised methods utilize human-annotated data to train networks to learn feature information hidden in images. Currently, state-of-the-art semantic segmentation methods employ deep learning methods for pixel-level prediction. U-Net has shown excellent performance in medical image segmentation due to its unique encoder-decoder structure. Many U-Net variants have been designed for retinal vessel segmentation. Jin et al. [5] proposed a method combining deformable convolution and U-Net to detect retinal blood vessels. Wu et al. [6] incorporated U-Net into a generative adversarial network. Retinal vessel segmentation is performed in an end-to-end manner. Although these methods have improved the accuracy of retinal vessel segmentation to a certain extent, the connectivity of vessels is difficult to guarantee due to insufficient use of contextual information in the structure, and the segmentation of thin vessels is still difficult. Clinically, thin blood vessels and vascular connectivity provide an indispensable reference for diagnosing vascular diseases. Therefore, it is imperative to explore new retinal vessel segmentation techniques.

To tackle the above-mentioned problem, this paper proposes an anatomy-sensitive retinal vessel segmentation framework that can jointly improve the performance of retinal vessel segmentation by exploiting the latent association among multiple modules. The backbone network adopts the improved U-Net network. To take full advantage of semantic information, we design a context relation module, which effectively combines the strong local modeling ability of convolution and the advantages of transformers in long-range modeling, and maps the features of various scales of the encoder to the decoder through skip pathways. In addition, we also design a sub-network for landmark detection, which learns a set of landmarks from retinal images using heatmap regression, to guide the network segmentation direction. The main contributions of this paper are as follows.

- TransUNet is more in line with anatomical retinal vessel segmentation due to its special structure. We use transformers as the segmentation backbone to benefit from the advantages of convolutional layers in extracting local features and multi-head self-attention in modeling global relations. Meanwhile, we reform the skip connections in TransUNet to decode deep semantics more easily and accurately.
- A self-supervised landmark-assisted segmentation framework is proposed to further improve the accuracy of retinal vessel segmentation. In particular, we propose a strategy for contrastive learning to improve the plausibility and accuracy of landmark representations of anatomical topology. We utilize landmarks that sparsely represent retinal vessel morphology to guide the model towards learning the content, rather than the style that is not conducive to segmentation. Furthermore, landmarks enhance the richness of explicit descriptions of retinal vascular anatomy, which is friendly for the model to learn based on fewer samples.
- We implement the proposed network on the DRIVE, CHASE-DB1, and STARE datasets, and extensive experimental results show that our method achieves state-of-the-art performance in most cases.

## 2. Related Work

Deep convolutional neural networks have become the most popular method for retinal vessel segmentation due to their excellent performance in medical image segmentation tasks. Among them, U-Net [7] and its variants are the most widely used as the backbone. A symmetric encoder-decoder structure and skip-connected architecture from encoding paths to decoding paths lead U-Net to achieve efficient information flow. Benefiting from an architecture that integrates local and global information from low-level and high-level feature maps, U-Net exhibits better performance in medical image analysis. However, although U-Net achieves multi-scale contextual information aggregation, it is still insufficient to cope with thin and irregular retinal vascular structures. Multiple studies have been devoted to addressing this issue.

Wang et al. [8] improved on the standard U-Net network and designed a two-channel encoder to extract information about retinal blood vessels. The improved encoder includes a context channel and a spatial channel to capture more receptive field and spatial information. The design of the backbone network of Li et al. [9] adopts the iterative principle to cascade multiple small U-Net networks to learn the structural features of retinal blood vessels. The input of each small U-Net network is the coarse segmentation probability map output by its previous U-Net network, and the vessel segmentation accuracy is improved by iterating from coarse to fine. Despite the excellent representational power of convolution, CNN-based methods often exhibit limitations in modeling explicit long-term relationships because of the inherent locality of convolution operations. The transformer module shows outstanding performance in capturing long-distance dependencies in the field of natural language processing, and is gradually being introduced into image processing. Cao et al. [10] designed the Swin-Unet network for medical image segmentation. The proposed network adopts a symmetrical structure similar to the U-Net network, and both the encoder and the decoder use pure transformer modules. However, the construction of a pure transformer network requires a large amount of computation, and the network is difficult to train. Xia et al. [11] proposed a combined CNN and transformer method to segment the optic cup and optic disc in the retina. First, the local features of the retina are obtained by convolution, and the extracted features are respectively passed through the multi-scale convolution module and the transformer module to obtain multi-scale feature information and global feature information. Finally, the segmentation performance of the optic cup and optic disc can be improved by fusing these two parts of the feature information. Chen et al. [12] integrated the transformer module into the U-Net network to achieve multi-organ segmentation. Convolutions are first utilized to extract low-level features, and then global interactions are modeled through the transformer module. The framework effectively combines the powerful local modeling capabilities of convolutions and the advantages of transformers in long-range modeling, enabling finer organ detail segmentation.

Accurate detection of landmark points is a critical step in medical imaging, as it provides quite valuable information for subsequent medical image analysis. Coordinate regression is the most typical method. The landmark coordinates are used as the target for the network regression to predict a set of landmark locations directly from the image space. Sun et al. [13] proposed a cascade of deep convolutional networks to improve the detection accuracy of face landmarks through coarse-to-fine regression. Zhang et al. [14] combined multi-task learning with a regression model for face landmark detection and used cascaded deep convolutional networks to predict face and landmark locations in a coarse-to-fine manner. However, the direct mapping from original images to landmark coordinates is a complex nonlinear problem that is not easily learned by the network. Compared with the numerical value of landmark coordinates, heatmaps can provide more abundant supervision information in space, which also improves the accuracy of landmark detection to a certain extent. Kowalski et al. [15] proposed a heatmap-based cascaded deep convolutional network DAN. The detected landmark positions are refined by each stage and passed to the next stage to correct the landmark positions iteratively. Shi et al. [16] designed a superimposed hourglass network and introduced offset learning to refine the predicted landmarks. The network effectively combines heatmap information and coordinate information to achieve accurate facial landmark detection.

Our proposed method focuses on improving the ability of the model to learn anatomical structures, thus achieving higher segmentation accuracy.

## 3. Methods and Materials

**Figure 1:**
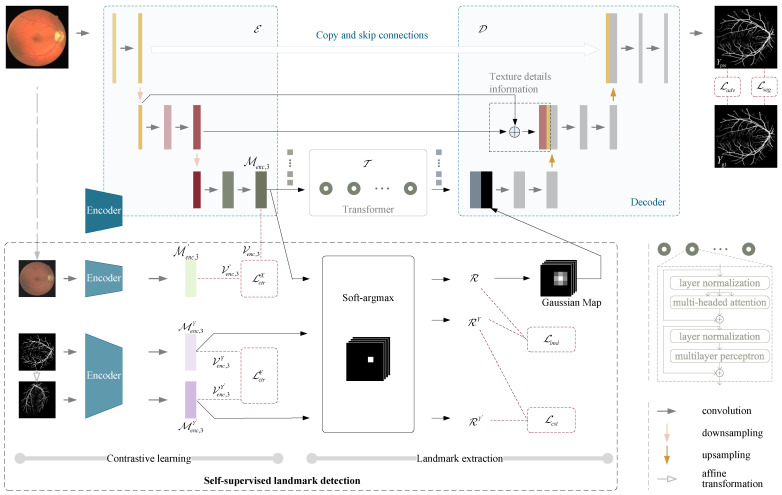
The pipeline of the proposed method.

Our objective is to develop a deep learning model for segmenting blood vessel pixels in retinal images. To achieve this, we propose a framework, as depicted in Figure 1, which comprises two main sections: (i) An enhanced version of the U-Net is employed for precise segmentation of fundus blood vessels. (ii) Additionally, landmark detection is used as an auxiliary task to further enhance the accuracy of segmentation.

### 3.1. Datasets

We use three public datasets for experiments, namely DRIVE, CHASE-DB1, and STARE. To improve the accuracy of segmentation, we implemented a data augmentation technique that utilized random flipping, rotation, and scaling.

The DRIVE dataset includes 40 fundus retinal color images, 7 of which are pathologically abnormal. The dimensions of each image are 584×565 pixels. The last twenty images of this dataset are used to train the network, and the first twenty images are used to test the network. All images in the test set consist of the results of manual segmentation by two professionals. We chose to use the result of the first professional manual segmentation as the label of the retinal blood vessels.

The CHASE-DB1 dataset contains 28 retinal images. They were taken from the eyes of 14 children. All images in the dataset are 996×960 pixels. Unlike the DRIVE dataset, there are no fixed training and test set partitions for CHASE-DB1. We randomly placed 20 retinal images in the training set and 8 images in the test set.

The STARE dataset has a total of 20 images. All images are 700×605 pixels. Since the STARE dataset does not have a pre-separated training set and test set, we employed leave-one-out cross-validation to verify the feasibility of our proposed method.

We improved upon the common approach of completely random data augmentation. First, we defined a sliding window with dimensions 0.6 times the width and height of the original image (i.e., the window area is 0.36 times that of the original image). Then, using this sliding window, we extracted 9 slices of the image with a stride of 1. Next, we selected the slice with the highest proportion of foreground from these 9 slices and performed other operations (such as flipping, contrast adjustment, brightness modification) before adding it to the training data. This approach helps to alleviate the issues of class imbalance or foreground–background imbalance to some extent.

### 3.2. TransUNet

Medical images have the unique advantage of having explicit contextual priors, due to the anatomical properties of tissues. Therefore, we propose to consider the long-range dependencies of pixels while also extracting local features. As illustrated in Figure 1, we introduce a transformer into the U-Net architecture. The convolutional layer of U-Net ensures that the model remains locally sensitive to the image, while the transformer module allows the model to capture global features of the image.

First, some symbols are defined. The convolutional encoder is $E={\left\{E_{\frac{H}{2^{n_{d}}}\times \frac{W}{2^{n_{d}}}}\right\}}_{n_{d}=0}^{N_{d}}$ where *H* and *W* are the height and width of the input of the convolution operator, respectively. $N_{d}$ is the number of down-sampling operations $f_{d}$. That is, convolution operators are grouped by the resolution of their input. Similarly, the convolutional decoder is $D={\left\{D_{\frac{H\cdot 2^{n_{u}}}{2^{N_{d}}}\times \frac{W\cdot 2^{n_{u}}}{2^{N_{d}}}}\right\}}_{n_{u}=0}^{N_{u}}$. The transformer module is denoted by T. The feature map is denoted by M with channel *C*.

### 3.3. Convolutional Encoder

The convolutional encoder of our method is the same as that of the standard U-Net encoder. Considering the missing information of tiny blood vessels caused by down-sampling and the over-fitting problem caused by too deep model layers, $N_{d}$ is set to 2. That is, the convolution operators are divided into three groups, i.e., the feature maps have three resolutions. The original image is denoted as *X*. Then,

(1) $$\begin{array}{cc} \; & M_{enc,1}=E_{H\times W}\left(X\right)\in R^{H\times W\times C_{1}},\\ \; & M_{enc,2}=E_{\frac{H}{2}\times \frac{W}{2}}\left(f_{d}\left(M_{enc,1}\right)\right)\in R^{\frac{H}{2}\times \frac{W}{2}\times C_{2}},\\ \; & M_{enc,3}=E_{\frac{H}{4}\times \frac{W}{4}}\left(f_{d}\left(M_{enc,2}\right)\right)\in R^{\frac{H}{4}\times \frac{W}{4}\times C_{3}}. \end{array}$$

### 3.4. Transformer Module

To address the challenges of training transformers and their resource-intensive nature, we propose to connect the transformer module behind the convolutional encoder. This approach allows the transformer to receive input of smaller resolution, thereby reducing the equipment resources required. Moreover, the feature maps that are fed into the transformer already contain deep semantic information, making it easier to train. By incorporating the transformer module in this way, we can ensure that the model captures both local and global information, as the transformer mines long-range dependencies based on feature maps that have already extracted local features.

First, $M_{enc,3}$ is decomposed into ${N_{P}}^{2}$ patches, i.e., $M_{enc,3}\mapsto {\left\{M_{enc,3}^{n_{P}}\in R^{\frac{H}{N_{P}}\times \frac{W}{N_{P}}\times C_{3}}\right\}}_{n_{P}=1}^{N_{P}}$. The input of the transformer is

(2) $$Z_{0}={\left\{z_{pos}^{n_{p}}+z_{pat}^{n_{p}}\right\}}_{n_{p}=1}^{{N_{p}}^{2}},$$

where $z_{pos}^{n_{p}}$ and $z_{pat}^{n_{p}}$ are the position embedding and feature embedding of $M_{enc,3}^{n_{P}}$, respectively. $z_{pat}^{n_{p}}=f_{pf}\left(M_{enc,3}^{n_{P}}\right)$, where $f_{pf}$ is patch-wise flatten.

The transformer module T is composed of $N_{t}$ transformer layers; each of them $T_{n_{t}}$ consists of a multi-head self-attention (MHSA) block, multi-layer perceptron (MLP) block, and layer normalization (LN) blocks. The output of the $n_{t}$-th transformer layer is $Z_{n_{t}}=T_{n_{t}}\left(Z_{n_{t}-1}\right)$, specifically,

(3) $$\begin{array}{cc} \; & Z_{n_{t}}^{{}^{\prime }}=MHSA\left(LN\left(Z_{n_{t}-1}\right)\right)+Z_{n_{t}-1},\\ \; & Z_{n_{t}}=MLP\left(LN\left(Z_{n_{t}}^{{}^{\prime }}\right)\right)+Z_{n_{t}}^{{}^{\prime }}. \end{array}$$

Finally, the output sequence $Z_{N_{t}}$ of T is reconstructed into $M_{t}$ by the patch merging layer $f_{pm}$, i.e., $M_{t}=f_{pm}\left(Z_{N_{t}}\right)\in R^{\frac{H}{4}\times \frac{W}{4}\times C_{3}}$.

### 3.5. Convolutional Decoder

We elaborately design a convolutional decoder D for progressive decoding. Similar to the convolutional encoder, the convolutional decoding layer is also divided into three groups according to the resolution, i.e., $N_{u}=2$. Up-sampling $f_{u}$ is bilinear interpolation. $D_{\frac{H}{4}\times \frac{W}{4}}$ is fed by $M_{dec,0}$ channel-wise connected by $M_{t}$ and $M_{gm}$, where $M_{gm}$ is the Gaussian map of the landmark. In a traditional UNet decoder, $D_{\frac{H}{2}\times \frac{W}{2}}$ is fed by the channel-wise connection of $f_{u}\left(D_{\frac{H}{2}\times \frac{W}{2}}\left(M_{dec,0}\right)\right)$ and $M_{enc,2}$. Considering the local detailed information lost due to the transformer modeling global relations, we further fuse $f_{d}\left(M_{enc,1}\right)$, which contains more texture information for $D_{\frac{H}{2}\times \frac{W}{2}}$. In particular, we enhance the sensitivity of convolutional encoders to anatomical topology through contrastive learning; $M_{enc,1}$ is considered to represent dense local shape details. In this way, when the global information and local information are fused in $D_{\frac{H}{2}\times \frac{W}{2}}$, they are constrained by the texture information of the shape, which can avoid decoding information that violates the anatomical topology. Formalized,

(4) $$\begin{array}{cc} \; & M_{dec,1}=D_{\frac{H}{4}\times \frac{W}{4}}\left(M_{t},M_{gm}\right)\in R^{\frac{H}{4}\times \frac{W}{4}\times C_{3}}.\\ \; & M_{dec,2}=D_{\frac{H}{2}\times \frac{W}{2}}\left(f_{u}\left(M_{dec,1}\right),M_{enc,2},f_{d}\left(M_{enc,1}\right)\right)\\ \; & \in R^{\frac{H}{2}\times \frac{W}{2}\times C_{2}},\\ \; & Y_{pre}=D_{H\times W}\left(f_{u}\left(M_{dec,2}\right),M_{enc,1}\right)\in R^{H\times W\times 1}, \end{array}$$

where $Y_{pre}$ is the label predicted by the model.

### 3.6. Self-Supervised Landmark Detection

To address the difficulty of segmenting thin blood vessels from the background in retinal images due to their high complexity and low contrast, we propose a novel approach that incorporates landmark points to assist the network in segmentation. This approach represents a departure from previous methods that relied solely on implicit feature vectors learned from images by the network for pixel-by-pixel segmentation. The introduction of landmark detection represents a critical component of our segmentation network. Considering the small amount of retinal vessel data and the high cost of manual annotation, we propose unsupervised learning of a set of ordered landmarks from dense retinal vessel images under the framework of contrastive learning to guide the model for segmentation. The detected landmarks sparsely characterize the key features of dense anatomical topology and thus can represent the intrinsic structure of fundus vessels. For the model to extract accurate and robust landmark points, we propose a contrastive learning strategy and introduce a series of optimization objectives to train the model. Landmarks are generated based on the heatmap of the convolutional encoder, as shown in the landmark detector part of Figure 1.

#### Coordinate Extraction for Landmarks

We extract the landmarks in a way that activates the highest weighted pixel in the feature map. We extract landmarks in the feature space $M_{enc,3}^{*}$ spanned by E. First, we adopt the spatial softmax normalization method to convert all channels of $M_{enc,3}^{*}$ to the probability response map $M_{prob}^{*}$. Then, the site with the highest weight in the probability map $M_{prob}^{*}$ is activated by soft-argmax as a landmark. Formally, the feature map $M_{enc,3}^{*,}\left[c\right]$ of the *c*-th channel is probabilized as

(5) $${\left.M_{prob}^{*}\left[c\right]=\frac{\exp(M_{enc,3}^{*,}\left[c,r\right])}{\sum_{r\in (\frac{H}{4}\times \frac{W}{4})}\exp(M_{enc,3}^{*,}\left[c,r\right])}\right|}_{r=1}^{\frac{H}{4}\times \frac{W}{4}}.$$

The set of landmark coordinates is

(6) $$R^{*}={\left\{soft-argmax(M_{prob}^{*}\left[c\right])\right\}}_{c=1}^{C_{3}},$$

where $R^{*}$ is the landmarks.

We utilize consistency loss $L_{cst}$ to guarantee the quality of landmarks. $L_{cst}$ is defined as

(7) $$L_{cst}=dist_{cst}\left(R^{Y},A_{Y}^{-1}\left(R^{Y^{\prime }}\right)\right),$$

where $dist_{cst}$ is the L2 distance. The landmarks are stable and reliable when the landmarks extracted in $Y^{{}^{\prime }}$ can be consistent with the landmarks extracted in *Y* by inverse affine transformation. This is as described in [17].

### 3.7. Landmark Auxiliary Guided Segmentation

The total loss for model training is

(8) $$L_{total}=\lambda_{1}L_{seg}+\lambda_{2}L_{adv}+\lambda_{3}\left(L_{ctr}+L_{cst}\right)+\lambda_{4}L_{lmd},$$

where $\lambda_{1}$, $\lambda_{2}$, $\lambda_{3}$ and $\lambda_{4}$ are the balance coefficients of corresponding loss. $L_{seg}$ is the pixel-level loss for the segmentation task.

(9) $$L_{seg}=BCE\left(Y_{pre},Y\right)+DICE\left(Y_{pre},Y\right)$$

where BCE and DICE are the binary cross-entropy (BCE) loss and dice loss, respectively. $L_{adv}$ is the adversarial loss as the global loss for segmentation.

(10) $$L_{adv}=E_{Y}\left[\log D(Y)\right]+E_{Y^{{}^{\prime }}}\left[\log(1-D\left(Y^{{}^{\prime }}\right))\right]$$

where D is the discriminator. $L_{seg}$ and $L_{adv}$ constrain the segmentation of the model locally and globally, respectively. $L_{lmd}$ is the landmark-based auxiliary loss based on optimal transport theory. We use the obtained landmarks based on ground truth *Y* as pseudo-labels, i.e., $R^{Y}$. $L_{lmd}$ is defined as

(11) $$L_{lmd}={∥R-R^{Y}∥}_{2}^{2}$$

where R is the landmark-obtained base on *X*. $L_{lmd}$ can guide the convolutional encoder to learn more effective information.

Further, we map the landmark information into a Gaussian map $M_{gm}$ that is easier to embed in the network, and feed it to the convolutional decoder in order to boost the performance of the decoder. The Gaussian map is defined as

(12) $$M_{gm}=\exp\left(-\frac{1}{2\sigma^{2}}{∥R-R^{Y}∥}^{2}\right),$$

where the standard deviation σ is set to 0.7 for all the experiments. Then, $M_{gm}$ is connected with $M_{t}$ channel-wise as the input of D.

As D accepts the input composed of $M_{t}$ and $M_{gm}$, it benefits from both global and local high-level semantic information extracted earlier. In particular, $M_{gm}$ is an explicit sparse representation of the anatomical topology. Additionally, $M_{gm}$ is a further disentangled representation of the anatomical topology. In addition, $M_{gm}$ provides the model with prior topological constraints, which enrich the semantics of the data.

### 3.8. Implementation Details

Considering that there are many tiny blood vessels in the retinal vascular structure, excessively deep convolutional layers may cause some features that are beneficial for segmentation to be ignored. Therefore, we only take the first three layers of the U-Net network and integrate the transformer module into the network in the third layer. Apart from that, the encoders in the semantic segmentation network share the same weights as those under the contrastive learning framework. After conducting experiments, we determined that the values of $\lambda_{1}$, $\lambda_{2}$, $\lambda_{3}$ and $\lambda_{4}$ should be set to 0.2, 0.3, 0.4, and 0.1, respectively.

During the training, instead of patches, we input the entire image into the model to generate the retinal vessel prediction map. We adopt Adam to optimize the deep model with an initial learning rate of 0.001 and a weight decay of 0.0005. Due to GPU memory constraints, we only input one retinal vessel image per iteration and resize all training images to 512×512 pixels. All models used in the experiments are implemented using pytorch-based python programs. They run on a computer configured with RTX3090 GPU.

## 4. Results and Discussions

### 4.1. Evaluation Metrics

The retinal vessel segmentation problem can be viewed as a binary classification. All pixels in retinal images can be classified into vascular and non-vascular pixels. Therefore, four definitions are derived according to the classification results of blood vessels. Those correctly classified as vascular pixels are regarded as true positives (TP). Those correctly detected as non-vascular pixels are counted as true negatives (TN). Those misclassified as non-vascular pixels are recorded as false positives (FP). Non-vascular pixels falsely detected as vascular pixels are counted as false negatives (FN).

To validate the feasibility of our designed network, we introduce four metrics of accuracy (Acc), sensitivity (Se), specificity (Sp), and F1 score to evaluate our network. Among them, the F1 score, as a trade-off between sensitivity and specificity, dominates the performance evaluation.

### 4.2. Comparison with the State-of-the-Art Methods

#### 4.2.1. Quantitative Analysis

We compare our method with other state-of-the-art methods on the DRIVE, CHASE, and STARE datasets. The experimental evaluation indicators are shown in Table 1. It is evident that our method achieves leading F1 scores in all three datasets. For the DRIVE dataset, the Se, Sp, Acc, and F1 scores obtained by our proposed method are 0.9577, 0.8147, 0.9862, and 0.8329, respectively. Jiang et al.’s method [18] obtained the highest Acc and Sp scores, but only 0.7839 and 0.8246 for Se and F1. These are far lower than our results, and our Sp is only 0.0028 lower than theirs, which can be negligible. In the CHASE dataset, we obtain Acc, Se, Sp, and F1 of 0.9754, 0.8110, 0.9881, and 0.8222, respectively. The best performance metrics obtained by other methods are 0.9670, 0.8329, 0.9813, and 0.8191, respectively. In contrast, our F1 reaches the peak of existing methods. Although the Acc, Se and Sp scores produced by our network are not optimal, these three metrics are also at high levels compared with other methods. On the STARE dataset, our method achieves high Acc, Sp, and F1 results while maintaining the highest Se score. Compared with other methods, these results show that our network has stronger vessel detection ability and stronger generalization ability across different databases.

#### 4.2.2. Qualitative Analysis

**Figure 2:**
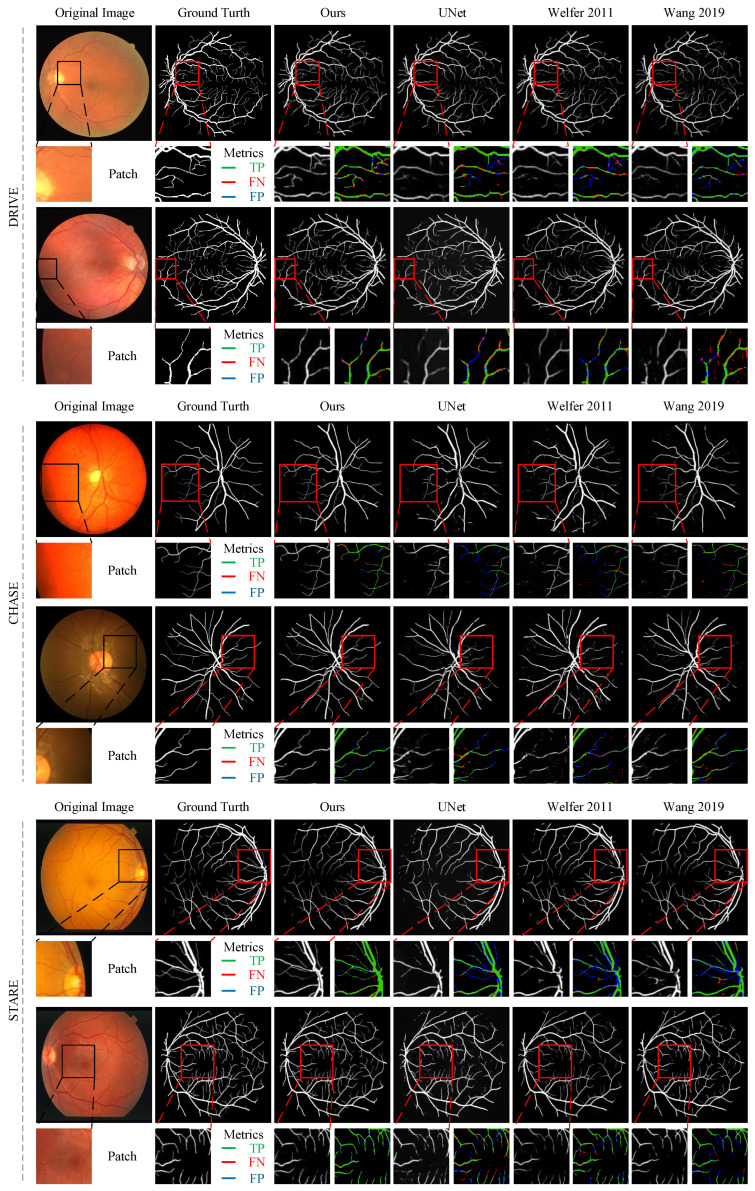
Examples of retinal vessel segmentation for three datasets (Welfer 2011 [4]; Wang 2019 [27]).

Figure 2 shows the results of retinal vessel segmentation using several representative methods and our proposed method. The results show that our proposed method preserves almost all the structures of retinal vessels and guarantees the connectivity of the vessel tree. In addition, the model can clearly segment from the background thin blood vessels that cannot be segmented by other methods, especially at the retinal edge and vessel ends. To more clearly show the difference between the prediction results of other network models and our network model, we visualize the local segmentation results of the model and color-label the different segmentation cases. Blue pixels in the image represent false negatives from undetected vessel regions. Red pixels represent false positives, indicating over-segmentation of blood vessels. It is evident from the patches in Figure 2 that the predicted segmentation maps of other methods show more blue pixels. This further proves that our proposed model has certain advantages in detecting thin blood vessels.

**Figure 3:**
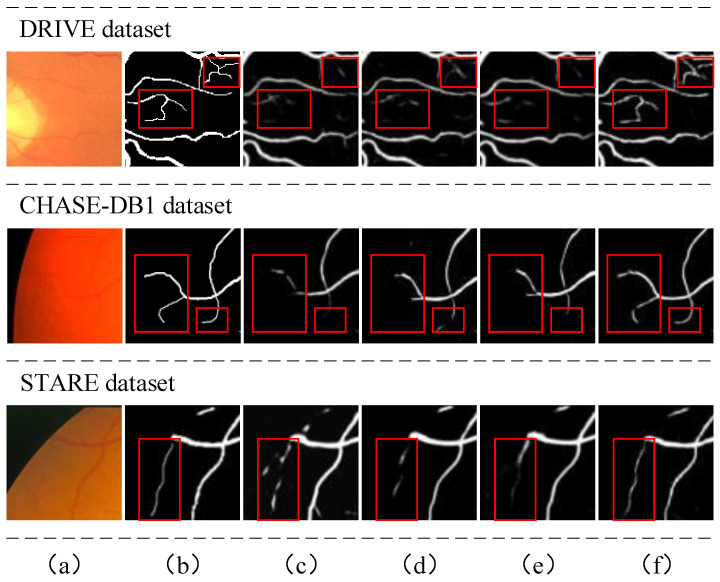
Locally magnified view of the segmentation results: (**a**) raw fundus image, (**b**) ground truth, (**c**) U-Net, (**d**) Jin 2019 [5], (**e**) Zhou 2020 [28], (**f**) our method.

Some segmentation examples are given in Figure 3, which contains locally enlarged images of the original retinal images, the corresponding ground truth values, and segmentation prediction maps obtained by several other methods and our proposed method. As can be seen from Figure 3, our algorithm can detect thin blood vessels more clearly and ensure connectivity between blood vessels.

These experimental data demonstrate that our model can more accurately distinguish vascular and non-vascular pixels and preserve vascular structure better.

### 4.3. Ablation Experiments

In this paper, we introduce the TransUNet structure and self-supervised landmark detection to improve retinal vessel segmentation performance. To test the effectiveness of these modules, ablation experiments are performed on DRIVE, STARE and CHASE-DB1. We start with the original U-Net method to evaluate how these modules affect segmentation performance. The self-supervised landmark detection is denoted by SLD. The results are shown in Table 2. For simplicity, we only visualize a few of the most representative instance images.

#### 4.3.1. Effect of TransUNet

**Figure 4:**
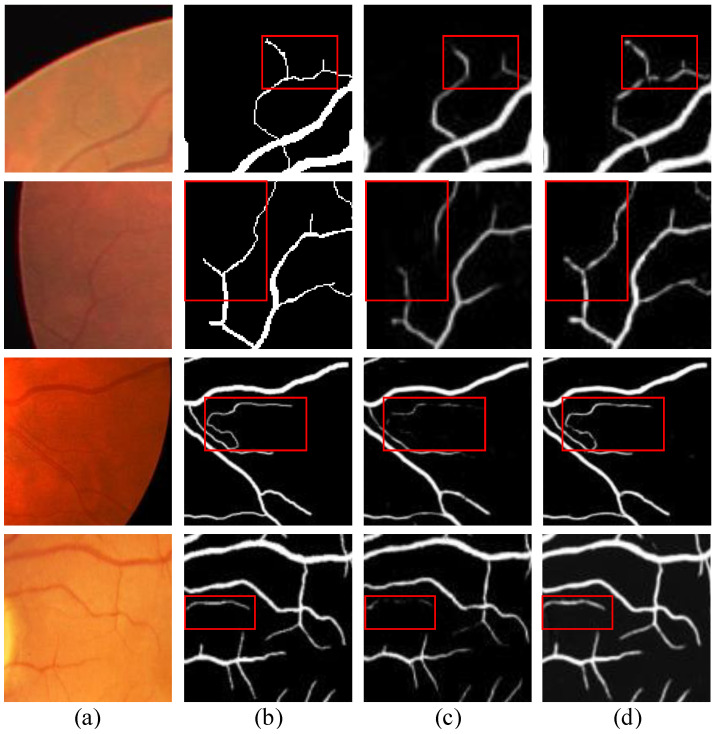
Illustration of vessel connectivity: (**a**) the retinal fundus patches, (**b**) ground truth, (**c**) segmentation output from U-Net, (**d**) segmentation output from TransUNet. First row and second row: DRIVE dataset, third row: CHASE-DB1 dataset, fourth row: STARE dataset.

To demonstrate the feasibility of the proposed TransUNet structure, we compare the U-Net network with the U-Net with transformer embedded. The same configuration and environment were used for both experiments. The results show that we achieve 0.9543, 0.7874, 0.9860, and 0.8148 on the DRIVE dataset for Acc, Se, Sp, and F1, respectively, and 0.9536, 0.7653, 0.9811, and 0.8078 on the baseline model for Acc, Se, Sp, and F1, respectively. At the same time, the performance on the other two datasets is also improved. Additionally, from the visualization in Figure 4, we can observe that the TransUNet structure can fully help the network to learn more feature information that ensures the connectivity of blood vessels.

#### 4.3.2. Effect of Self-Supervised Landmark Detection

**Figure 5:**
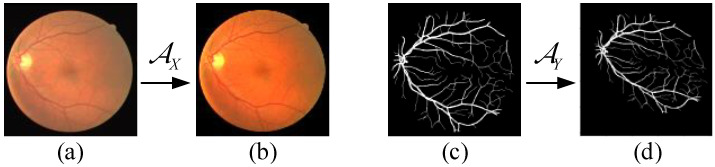
Example of transformation: (**a**) original retinal image, (**b**) style-transformed retinal image, (**c**) ground truth, (**d**) ground truth image after affine transformation.

To justify the use of landmark points to guide network segmentation, in Figure 5, we show an example visualization including the original retinal image and the style-transformed image, ground truth, and the affine-transformed ground-truth image of the DRIVE dataset.

The affine transformation matrix is shown in (13).

(13) $$A=\left(\begin{array}{ccc} 0.90411 & 0.17613 & 0\\ 0.05871 & 0.82583 & 0 \end{array}\right),$$

**Figure 6:**
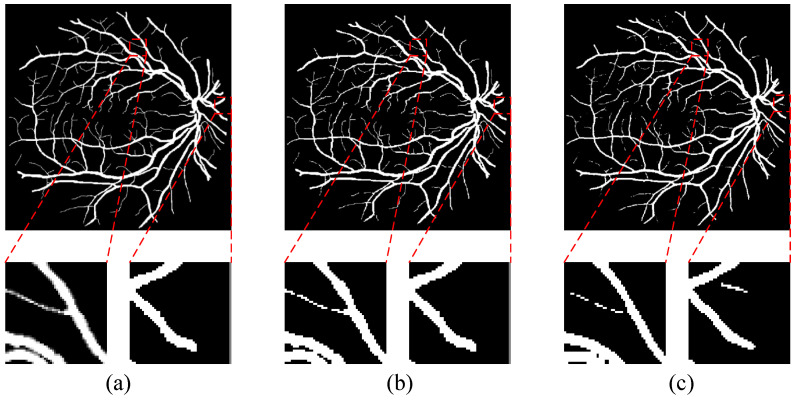
Illustration of thin vessel segmentation results: (**a**) ground truth, (**b**) segmentation results of the network without the self-supervised landmark detection module, (**c**) segmentation results of our method.

According to Table 2, it can be observed that the segmentation results with the addition of the self-supervised cues show improvements on all three datasets to varying degrees. Furthermore, in the visualization results shown in Figure 6, the segmentation guided by the self-supervised cues demonstrates superior performance in segmenting small blood vessels.

Therefore, our proposed landmark detection module can help us detect thin blood vessels more accurately.

### 4.4. Effect of Image Size

**Figure 7:**
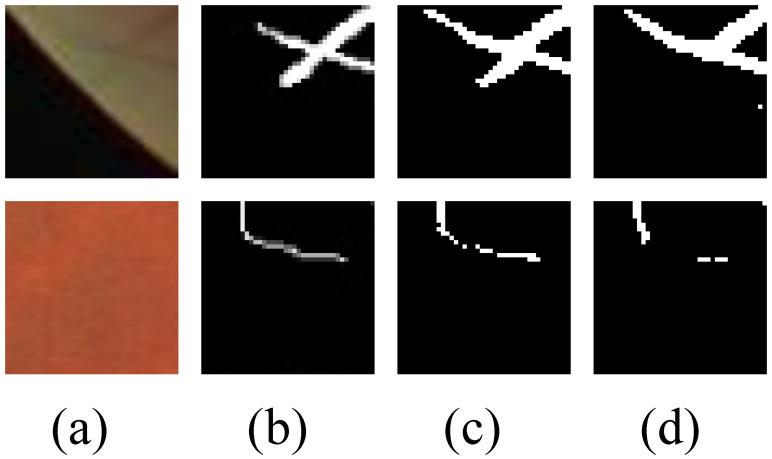
Sample segmentation results for small blood vessels in images of different sizes: (**a**) original retinal image, (**b**) ground truth, (**c**) segmentation results for an input image of size 256 × 256 pixels, (**d**) segmentation results for an input image of size 512 × 512 pixels.

As is customary in most works, we initially resized all training images to dimensions of 512 × 512 pixels. However, inspired by the findings in work [34] regarding the impact of image size on deep learning, we conducted an additional evaluation. We resized the images to a dimension of 256 × 256 pixels and performed training accordingly. As shown in Table 3, the adjusted F1 scores and other metrics exhibited improvements. Moreover, as illustrated in Figure 7, the visualizations demonstrate that the segmented vessels became more intact.

## 5. Conclusions

In this paper, we construct a novel retinal vessel segmentation framework, aiming to address the problems of vessel breakage and low accuracy of thin vessels in segmentation. The U-Net acts as the basic network. The designed TransUNet structure combines context information of different scales in the process of encoding and decoding, which effectively ensures the connectivity of blood vessels. The detected landmarks sparsely represent the anatomical features of retinal blood vessels, and segmentation guided by landmarks can help the network better detect thin blood vessels. Experimental results on three public datasets demonstrate that our constructed network outperforms the existing mainstream networks. In the future, we will conceive more methods to integrate into the retinal segmentation network.

## Figures and Tables

**Table 1:** Performance comparison with state-of-the-art methods on the DRIVE, CHASE-DB1 and STARE datasets.

		DRIVE				CHASE-DB1				STARE			
**Method**	**Year**	**Acc**	**Se**	**Sp**	**F1**	**Acc**	**Se**	**Sp**	**F1**	**Acc**	**Se**	**Sp**	**F1**
U-Net [7]	2015	0.9536	0.7653	0.9811	0.8078	0.9604	0.7870	0.9777	0.7828	0.9588	0.7639	0.9796	0.7817
Orlando et al. [19]	2017	0.9454	0.7897	0.9684	0.7857	0.9467	0.7565	0.9655	0.7332	0.9519	0.7680	0.9738	0.7644
Zhang et al. [20]	2017	0.9466	0.7861	0.9712	0.7953	0.9502	0.7644	0.9716	0.7581	0.9547	0.7882	0.9729	0.7815
Srinidhi et al. [21]	2018	0.9589	0.8644	0.9667	0.7607	0.9474	0.8297	0.9663	0.7189	0.9502	0.8325	0.9746	0.7698
Yan et al. [22]	2018	0.9542	0.7653	0.9818	-	0.9610	0.7633	0.9809	-	0.9612	0.7581	0.9846	-
Xu et al. [23]	2018	0.9557	0.8026	0.9780	0.8189	0.9613	0.7899	0.9785	0.7856	0.9499	0.8196	0.9661	0.7982
Zhuang et al. [24]	2018	0.9561	0.7856	0.9810	0.8202	0.9536	0.7978	0.9818	0.8031	-	-	-	-
Alom et al. [25]	2019	0.9556	0.7792	0.9813	0.8171	0.9634	0.7756	0.9820	0.7928	0.9712	0.8292	0.9862	0.8475
Jin et al. [5]	2019	0.9566	0.7963	0.9800	0.8237	0.9610	0.8155	0.9752	0.7883	0.9641	0.7595	0.9878	0.8143
Jiang et al. [18]	2019	0.9709	0.7839	0.9890	0.8246	0.9721	0.7839	0.9894	0.8062	0.9781	0.8249	0.9904	0.8482
Guo et al. [26]	2019	0.9561	0.7891	0.9804	0.8249	0.9627	0.7888	0.9801	0.7983	-	-	-	-
Wang et al. [27]	2019	0.9567	0.7940	0.9816	0.8270	0.9661	0.8074	0.9821	0.8037	-	-	-	-
Zhou et al. [28]	2020	0.9535	0.7473	0.9835	0.8035	0.9506	0.6361	0.9894	0.7390	0.9605	0.7776	0.9832	0.8132
Xu et al. [29]	2020	0.9557	0.7953	0.9807	0.8252	0.9650	0.8455	0.9769	0.8138	0.9590	0.8378	0.9741	0.8308
Wang et al. [30]	2020	0.9581	0.7991	0.9813	0.8293	0.9670	0.8329	0.9813	0.8191	0.9673	0.8186	0.9844	-
Li et al. [9]	2020	0.9573	0.7735	0.9838	0.8205	0.9760	0.7969	0.9881	0.8072	0.9701	0.7715	0.9886	0.8146
Mou et al. [31]	2021	0.9553	0.8154	0.9757	0.8228	0.9651	0.8329	0.9784	0.8141	0.9670	0.8396	0.9813	0.8420
Zhang et al. [32]	2022	0.9565	0.785	0.9618	0.82	-	-	-	-	0.9668	0.8002	0.9864	0.8289
Liu et al. [33]	2023	0.9561	0.7985	0.9791	0.8229	0.9672	0.8020	0.9794	0.8236	0.9635	0.8039	0.9836	0.8315
Proposed	2023	0.9577	0.8147	0.9862	**0.8329**	0.9754	0.8110	0.9881	**0.8222**	0.9635	0.8518	0.9829	**0.8450**

**Table 2:** Ablation studies on the DRIVE, CHASE-DB1 and STARE datasets.

	DRIVE				CHASE-DB1				STARE			
**Method**	**Acc**	**Se**	**Sp**	**F1**	**Acc**	**Se**	**Sp**	**F1**	**Acc**	**Se**	**Sp**	**F1**
U-Net	0.9536	0.7653	0.9811	0.8078	0.9604	0.7870	0.9777	0.7828	0.9588	0.7639	0.9796	0.7817
TransUNet	0.9543	0.7874	0.9860	0.8148	0.9681	0.7994	0.9878	0.8079	0.9610	0.7670	0.9879	0.8057
TransUNet + SLD	0.9577	0.8147	0.9862	0.8329	0.9754	0.8110	0.9881	0.8222	0.9635	0.8518	0.9829	0.8450

**Table 3:** Segmentation results for images of different sizes on the DRIVE, CHASE-DB1 and STARE datasets.

	DRIVE				CHASE-DB1				STARE			
**Size**	**Acc**	**Se**	**Sp**	**F1**	**Acc**	**Se**	**Sp**	**F1**	**Acc**	**Se**	**Sp**	**F1**
512 × 512	0.9577	0.8147	0.9862	0.8329	0.9754	0.8110	0.9881	0.8222	0.9635	0.8518	0.9829	0.8450
256 × 256	0.9688	0.8188	0.9869	0.8455	0.9685	0.8155	0.9889	0.8243	0.9641	0.8188	0.9888	0.8466

## Data Availability

No additional data are available.
